# A Single-Magnet-Driven Low-Frequency Piezoelectric–Electromagnetic Hybrid Energy Harvester with Magnetic Coupling for Self-Powered Sensors

**DOI:** 10.3390/s26092757

**Published:** 2026-04-29

**Authors:** Shuaiting Chen, Minglei Han, Weian Wang, Chen Ren, Shuangbin Liu

**Affiliations:** 1 School of Mechanical and Vehicle Engineering, Changchun University, Changchun 130022, China; 2 School of Mechanical and Aerospace Engineering, Jilin University, Changchun 130025, China

**Keywords:** hybrid energy harvester, low-frequency energy conversion, human motion, electromagnetic effect, piezoelectric effect

## Abstract

Vibration energy is widely present in the natural environment. In the development of wearable self-powered systems, how to efficiently harvest the low-frequency mechanical energy of human motion has always been a core challenge. The piezoelectric–electromagnetic hybrid energy harvester designed in this paper consists of two units: a piezoelectric unit and an electromagnetic unit. The piezoelectric unit is composed of two arched plates, a piezoelectric layer, and an end magnet. The two sides of the piezoelectric unit are completely symmetrical. The electromagnetic unit is composed of a hollow tube, a central magnet, and a coil. The coil is wound around the outside of the center of the hollow tube to ensure that the central magnet can cut more magnetic flux lines. The two units output voltage through an external load. Firstly, based on a physical model, the force–electricity coupling mechanism is derived, and the dynamic response of the harvester at different frequencies is systematically tested. Secondly, through simulation and experiment, the influencing factors of the output voltage are deeply studied, and it is concluded that at medium and low frequencies (5 Hz–15 Hz), the harvester can provide efficient voltage output. The electromagnetic unit dominates at low frequencies and can output a larger voltage, but the voltage drops significantly after a certain frequency. The piezoelectric unit can supplement after the electromagnetic voltage drops, and the two have a synergistic effect. In addition, the output characteristics of the system mainly depend on frequency, initial distance, coil turns, and magnet mass. This paper clarifies the inherent physical mechanism of the hybrid energy harvester and provides an effective scientific reference for practical human motion energy conversion applications.

## 1. Introduction

In the era of rapid development of wearable devices and smart sensors, realizing self-powered systems has become an important direction for promoting the research of low-power electronic devices. Recent studies have shown that biomechanical energy from foot strike, joint motion and limb swing is a promising power source for wearable and implantable electronics, especially for long-term sensing and health monitoring [1,2,3,4,5]. Among many energy conversion mechanisms, piezoelectric energy harvesting technology [6,7,8,9,10] has been widely studied due to its simple structure and high durability. However, it has certain limitations in low-frequency environments, which limits the continuous output capability of devices in low-frequency environments [11,12]. Moreover, in long-term use, piezoelectric materials will fail or even break due to fatigue strength accumulation [13]. Beeby et al. [14] pointed out that although the service life of piezoelectric materials can be extended under compressive strain conditions, the stringent requirements on strain direction and application method greatly limit the structural form and practical application scenarios of the harvester. From MEMS-scale structures to flexible nanogenerators, piezoelectric energy harvesters have developed rapidly in materials, structural design and application scenarios, making them one of the most mature routes for self-powered systems [15,16,17,18]. Electromagnetic energy harvesting [19,20,21,22,23] uses the interaction between the coil and the magnetic field to generate current through relative motion. The main advantage of this technology is that it can work in a wider frequency range, especially suitable for medium- and low-frequency vibration environments. However, its core bottleneck is the difficulty of miniaturization, and once the system size is reduced, the magnetic flux drops precipitously. Therefore, the traditional single physical mechanism is difficult to consider the system efficiency and environmental adaptability. Recent electromagnetic harvesters have further improved low-frequency performance through magnetic plucking, dual-resonance design and flutter-induced motion, showing the continuing value of electromagnetic conversion for medium- and low-frequency environments [24,25,26,27].

To address this challenge, a hybrid energy harvesting structure combining piezoelectric and electromagnetic effects is a promising and feasible solution [28,29]. Theory and experiments show that by generating voltage through magnetic field changes and electromagnetic induction, and introducing piezoelectric units, not only can the energy conversion capability of the harvester be significantly enhanced, but the operating bandwidth can also be effectively widened and the stability of the output power improved [30,31,32,33,34].

Yao and Li [35] proposed a piezoelectric–electromagnetic hybrid energy harvester based on a planetary gear system. The external excitation frequency can be amplified twice through gear transmission meshing to achieve higher energy output. Under the condition of eight permanent magnets and external excitation of 7 Hz, the output voltage can reach 10.4 V, and the average output power is 250 mW. Zărnescu et al. [36] proposed a hybrid piezoelectric harvester consisting of two disc magnets, one sliding cylindrical magnet, one coil, and a piezoelectric element assembly, which utilizes the advantage of multiple turns to travel with a peak voltage between 12 V and 30 V in the case of a 12 mm diameter magnet. Saboor et al. [33] proposed a wind energy harvester, which harvests vortex-induced vibration through a piezoelectric sheet and an electromagnet at the same time, and can be used for sensor power supply in low-speed environment detection. Farhan et al. [37] proposed a new piezoelectric–electromagnetic hybrid energy harvester. The harvester is designed with a cantilever beam, which can work cooperatively with the electromagnetism by using its dynamic characteristics. Under the action of high-frequency bandwidth, the comprehensive average output voltage is 27.824 V. Lv et al. [38] proposed a reciprocating piezoelectric–electromagnetic hybrid energy harvester based on low-frequency human motion. Under optimal parameters, the output power of the system is 19.84 mW, and the maximum power density is 0.132 mW mm^−3^. Su et al. [39] developed a piezoelectric–electromagnetic hybrid energy harvester with FUC mechanism; the maximum power can reach 26.4 mW at 4 Hz. Li et al. [40] designed a piezoelectric and electromagnetic hybrid energy harvester based on two-stage magnetic coupling; the increase in magnetic pole alternating frequency can improve the voltage and expand the system bandwidth, and the maximum voltage of 3.4 V can be obtained at 200–300 rpm.

Although various energy harvesting schemes have been proposed, the energy conversion efficiency of the existing hybrid energy harvesting devices is still low when responding to low-frequency band (such as human gait) excitation, and the adaptability to external random disturbance is poor. Nevertheless, many existing hybrid harvesters still face one or more of the following limitations: relatively high operating frequency, limited miniaturization, insufficient adaptability to irregular human-motion excitation, or incomplete discussion of how magnetic coupling simultaneously affects piezoelectric deformation and electromagnetic induction [25,26,27,41,42,43,44,45,46,47,48,49]. To address the inefficiency of traditional linear energy harvesters in low-frequency environments, this paper proposes a hybrid piezoelectric–electromagnetic energy harvester driven by a single magnet and based on nonlinear magnetic coupling. Unlike simply connecting two independent power-generating units in parallel, the core scientific novelty of this design lies in replacing traditional direct mechanical impact with structurally amplified nonlinear magnetic interactions. As the central magnet undergoes reciprocating motion under external excitation, it not only generates an induced electromagnetic current but also dynamically alters the magnetic repulsion force acting between itself and the end magnets. This nonlinear magnetic force is harvested and mechanically amplified by an arched plate, thereby effectively converting large-amplitude, low-frequency macroscopic displacements into concentrated microscopic strains within the piezoelectric layer. By integrating nonlinear magnetic dynamics with a structural force amplification mechanism, this work offers valuable insights for the field of biomechanical energy harvesting. This paper is organized as follows: Section 2 introduces the structure and physical model of the energy harvester; Section 3 analyzes the influence factors on voltage output through simulation; Section 4 presents experimental research; Section 5 describes the voltage that can be obtained in practical application; and Section 6 draws conclusions.

## 2. Physical Model and Working Principle

**Figure 1:**
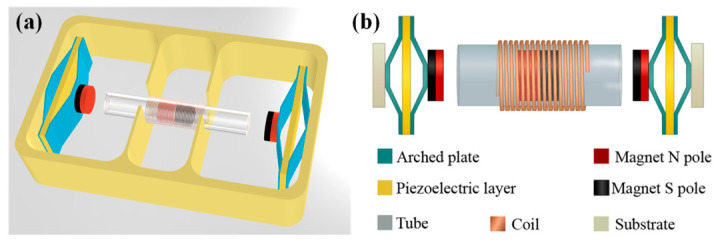
(**a**) Axonometric drawing; (**b**) top view.

As shown in Figure 1, this paper proposes a piezoelectric–electromagnetic hybrid energy harvester (PEHEH). The proposed PEHEH consists of two sets of flextensional piezoelectric units [50], a magnet attached to the end of an arched plate, a cylindrical central magnet located between the two end magnets, a hollow tube constraining the motion trajectory of the central magnet, an externally wound coil, and a substrate that withstands human motion excitation. The two sets of flextensional piezoelectric units and the hollow tube are fixed on the substrate. Each set of flextensional piezoelectric units is composed of a piezoelectric layer laminated with two inner and outer arched plates. The arched plates are formed by combining two sections of flat surfaces, two sections of inclined surfaces, and an upper flat surface, which are used to transmit and amplify the magnetic repulsion force. The central magnet, hollow tube, and external coil together constitute the electromagnetic harvesting unit. Thus, the harvester forms a composite structure centered around the central magnet and simultaneously coupled with both piezoelectric and electromagnetic energy conversion forms.

**Figure 2:**
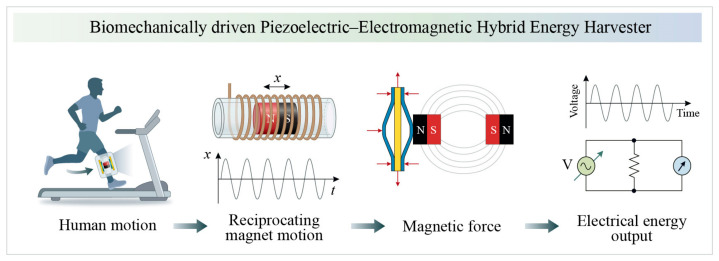
The working principle of the proposed PEHEH.

As shown in Figure 2, the working principle of the PEHEH can be summarized as follows. The vibration of the base caused by human motion is transmitted to the central magnet, causing it to reciprocate within the hollow tube. The diameter difference between the central magnet and the inner wall of the hollow tube is less than 0.5 mm. This small clearance is mainly used to suppress magnet rolling or severe tilting during reciprocation. In addition, the magnetic repulsion from the two end magnets keeps the central magnet within the hollow tube and maintains its guided reciprocating motion. The reciprocating motion of the central magnet relative to the coil continuously changes the magnetic field state near the coil, thereby generating an induced electromotive force in the coil circuit to achieve electromagnetic energy output. Simultaneously, the gap between the central magnet and the two end magnets also changes, causing a dynamic change in the magnetic repulsion force on both sides. This changing magnetic repulsion force is transmitted and amplified through the arched plate, causing a slight deformation of the piezoelectric layer and outputting piezoelectric energy. This energy harvester does not simply place two power generation units side by side, but rather simultaneously stimulates both piezoelectric and electromagnetic energy conversion mechanisms through the single movement of the central magnet. By synergistically harvesting energy using both piezoelectric and electromagnetic mechanisms, a more stable energy output can be achieved under low-frequency, broadband human movement excitation.

**Figure 3:**
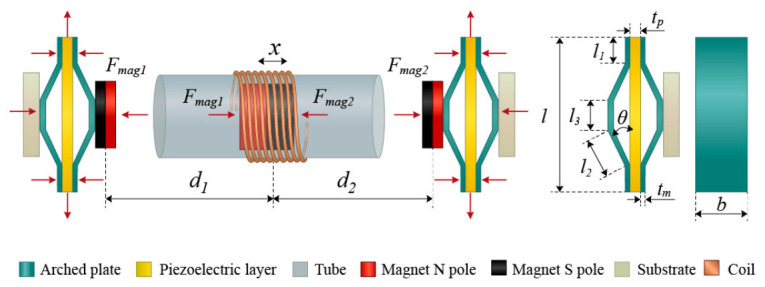
Force analysis and geometric parameters.

The force analysis and geometric parameters of the proposed energy harvester are shown in Figure 3. The dynamic equation of the electromagnetic system can be established as follows:

(1) $$\left\{\begin{array}{l} M_{c}\ddot{x}+D_{c}\dot{x}+\Theta I_{c}+F_{mc}+\lambda M_{c}g\cos\varphi =-M_{c}{\ddot{x}}_{base}\\ \theta \dot{x}+(R_{c}+r_{c})I_{c}-L_{c}{\dot{I}}_{c}=0 \end{array}\right.$$

(2) $$V_{1}=\frac{d_{\mathrm{eff}}}{C_{P}}F_{mag1}$$

(3) $$V_{2}=\frac{d_{\mathrm{eff}}}{C_{P}}F_{mag2}$$

Equation (1) is the dynamic equation of the electromagnetic element. Equations (2) and (3) represent the open-circuit voltage of the piezoelectric unit acquisition circuit [51]. In the formula, *M*_c_ represents the mass of the central magnet, *x* is the displacement of the central magnet, $\ddot{x}$ is the excitation acceleration, *D*_c_ is the electromagnetic damping coefficient, and *Θ* is the electromagnetic coupling coefficient of the coil. The magnetic force exerted on the *F*_mc_ centered magnet, *F_mc_ = F_mag_*_1_ + *F_mag_*_2_. It is related to the displacement between the central magnet and the endmost magnet. Subscripts 1 and 2, respectively, represent the left end magnet and the right end magnet; *V*_1_ and *V*_2_ are the output voltages of the piezoelectric layers on both sides, respectively. In the present study, λ is treated as an effective constant parameter representing the overall contact-related mechanical dissipation during reciprocation. Possible additional losses caused by slight eccentric contact are not resolved separately but are included in the effective friction term. g is the acceleration due to gravity; *φ* is the inclination angle of the hollow tube; I_C_ is the induced current generated by the coil; *R*_C_ is an electromagnetic external load resistor; *r*_c_ is the internal resistance of the coil; and *L*_c_ is the inductance of the coil.

The electromagnetic damping coefficient *D*_c_ is obtained by the vibration attenuation method, and the coil internal resistance *r*_c_ and inductance *L*_c_ can be directly measured by measuring instruments. *d*_eff_ represents the equivalent piezoelectric coefficient, which can be expressed as [52]

$$d_{eff}=-d_{33}+\frac{l_{2}^{3}(2l_{2}\cos\theta +l_{3})\sin\theta \cos\theta }{t_{p}l_{2}^{3}\sin^{2}\theta +3s_{11}D(2l_{2}\cos\theta +l_{3})}d_{31}$$

In the equation, *d*_31_ and *d*_33_ are piezoelectric constants; *l*_1_, *l*_2_, *l*_3_ are the lengths of the bonding plane, inclined plane, and upper plane, respectively, as shown in Figure 3; *θ* is the angle between the bonding plane and the inclined plane; *b* is the width of arched plate; *t*_p_ is the thickness of the piezoelectric layer; *s*_11_ is the elastic compliance of the piezoelectric layer; *D* is the bending stiffness of the metal layer, and *D* = *E*_m_*t*_m_^3^/12(1 − *v_m_*^2^), where *t*_m_ is the thickness of the arched plate, and *E*_m_ and *v*_m_ are the elastic modulus and Poisson’s ratio of the arched plate, respectively.

Magnetic forces *F_mag_*_1_ and *F_mag_*_2_ can be expressed as [51]

(4) $$F_{mag1}=\frac{3\mu_{0}M^{2}}{2\pi d_{1}^{4}}$$

(5) $$F_{mag2}=\frac{3\mu_{0}M^{2}}{2\pi d_{2}^{4}}$$

Among them, *μ*_0_ is the vacuum magnetic permeability, and *μ*_0_ = 4*π* × 10^−^^7^ Hm^−1^; *M* is the magnetic dipole moment; and *d*_1_ and *d*_2_ can be written as *d*_1_ = *d*_0_ + *x* and *d*_2_ = *d*_0_ − *x*, where *d*_0_ is the initial distance between magnetic dipoles.

## 3. Simulation Analysis

### 3.1. Simulation Analysis of the Influence of Initial Distance d_0_ on Piezoelectric and Electromagnetic Voltages

By changing the initial distance *d*_0_ between the end magnets at both ends and the central magnet, the influence of magnetic coupling strength on the dynamic response and energy output characteristics of the system is explored. The simulation parameters are 800 turns of coil and a 2.4 g (6 × 12) NdFeB magnet with N52 performance.

**Figure 4:**
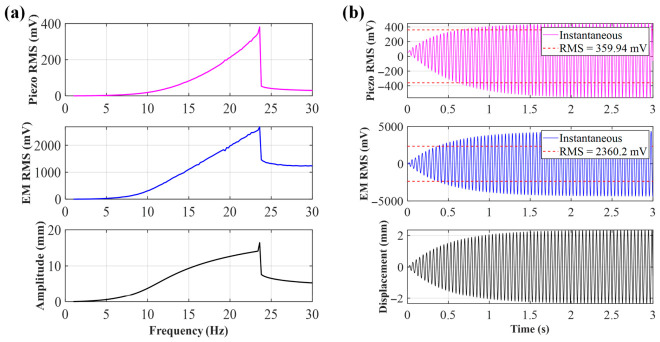
(**a**) Sweep voltage and amplitude of *d*_0_ = 25 mm; (**b**) voltage and displacement of *d*_0_ = 25 mm at 23.6 Hz.

When the initial distance *d*_0_ = 25 mm, due to the extremely small distance between the central magnet and the end magnet, the system is in a strong magnetic coupling state. As shown in Figure 4, at this distance, the voltages of the piezoelectric and electromagnetic units show a clear rising trend with increasing frequency, with spikes appearing at 23.6 Hz; the piezoelectric peak reaches 359.94 mV, and the electromagnetic peak reaches 2360.2 mV. Figure 4b shows the time-domain simulation at the peak frequency. The essence of this phenomenon lies in the magnetic repulsion between the magnets being inversely proportional to d4. The extremely strong magnetic repulsion causes the central magnet to move rapidly inside the hollow tube, leading to a sharp increase in the rate of change of magnetic flux, while the arched plate experiences significant bending stress, making the voltage changes generated by both the electromagnetic and piezoelectric units significant.

**Figure 5:**
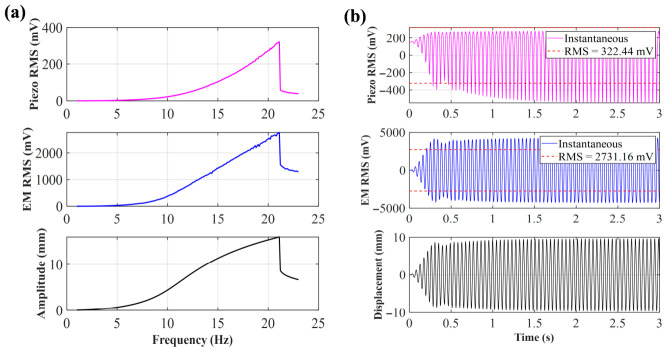
(**a**) Sweep voltage and amplitude of *d*_0_ = 30 mm; (**b**) voltage and displacement of *d*_0_ = 30 mm at 21.6 Hz.

As shown in Figure 5a, when the initial distance *d*_0_ = 30 mm, similar to *d*_0_ = 25 mm, although the distance between the central magnet and the end magnet increases, the system still belongs to a strong magnetic coupling state and maintains nonlinear dynamic characteristics. From the simulation results, it can be seen that the frequency of the peak voltage has decreased to around 21.6 Hz. This offset indicates that the significant nonlinear stiffness effect of magnetic repulsion on the system still exists. Under strong excitation, the electromagnetic and piezoelectric voltages can still exhibit significant jumps at the resonance point. However, due to the increase in initial distance, the magnetic repulsion decreases, and compared to *d*_0_ = 25 mm, the piezoelectric peak voltage decreases, but the electromagnetic peak voltage slightly increases. Figure 5b shows the time-domain simulation analysis of the frequency peak point. It can be seen from the figure that in the first 0.2 s, the displacement and voltage output are not significant under the action of vibration. After a short period of energy harvesting, the displacement of the central magnet increases, and then the system enters the periodic oscillation stage.

**Figure 6:**
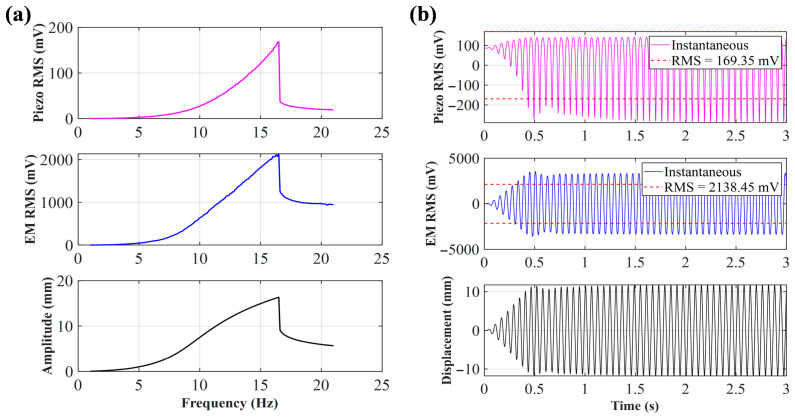
(**a**) Sweep voltage and amplitude of *d*_0_ = 33 mm; (**b**) voltage and displacement of *d*_0_ = 33 mm at 16.5 Hz.

As shown in Figure 6a, in the case of *d*_0_ = 33 mm, the nonlinear “cliff-like” drop phenomenon of the system is obvious. From the simulation results, the frequency of the peak voltage of the system is further shifted to the left, reaching 16.5 Hz. As shown in Figure 6b, at the steady-state resonance of 16.5 Hz, the electromagnetic output of the system is particularly strong, with an effective value (RMS) of 2138.45 mV, and the piezoelectric layer, relying on the mechanical coupling conduction of the arched plate, also outputs an effective voltage of 169.35 mV.

**Figure 7:**
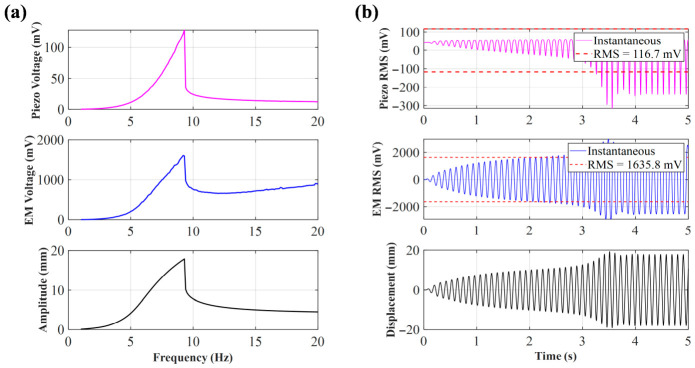
(**a**) Sweep voltage and amplitude of *d*_0_ = 40 mm; (**b**) voltage and displacement of *d*_0_ = 40 mm at 9.3 Hz.

As show in Figure 7, when *d*_0_ is expanded to 40 mm, the system reaches a relatively ideal equilibrium state. The frequency sweep simulation shows that the peak frequency has significantly decreased to around 9.3 Hz, perfectly matching the original intent of this article’s design—to achieve efficient energy harvesting in low-frequency environments (such as low-frequency movements like human walking). Although the peak voltage (piezoelectric about 115 mV, electromagnetic about 1607 mV) is significantly reduced compared to narrow spacing, the system can still trigger effective energy conversion using a nonlinear hopping mechanism at low frequencies, proving that this initial magnetic spacing is one of the ideal operating points for balancing low-frequency response.

When the initial distance *d*_0_ increases to 49 mm, the coupling between the magnets rapidly weakens and can even be ignored. The simulation curve shows that within the conventional frequency range of 0 Hz to 20 Hz, the piezoelectric and electromagnetic voltages only exhibit an extremely slow linear upward trend, completely losing the peak frequency and jump phenomenon mentioned in the previous section. In addition, the magnetic flux change rate and piezoelectric deformation are severely insufficient, resulting in extremely low energy output across the entire frequency band.

**Figure 8:**
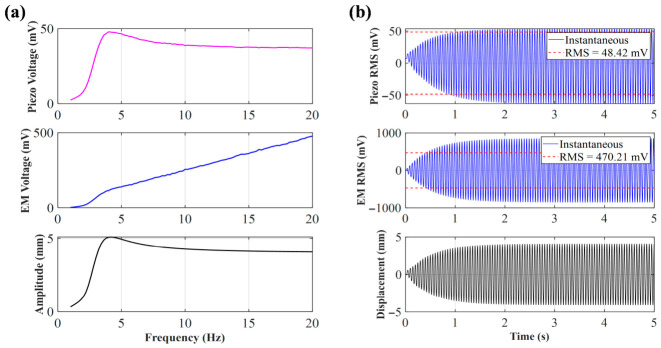
(**a**) Sweep voltage and amplitude of *d*_0_ = 49 mm; (**b**) voltage and displacement of *d*_0_ = 49 mm at 20 Hz.

**Figure 9:**
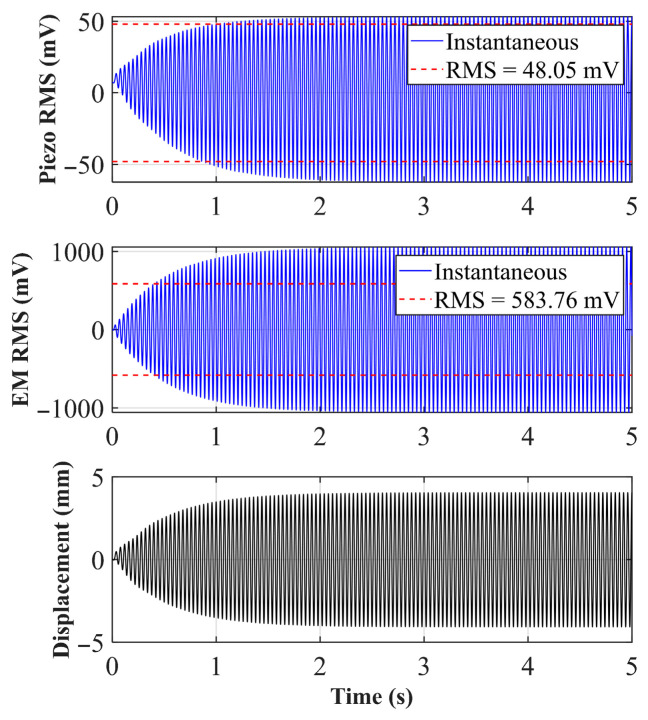
Voltage and displacement of *d*_0_ = 49 mm at 25 Hz.

**Figure 10:**
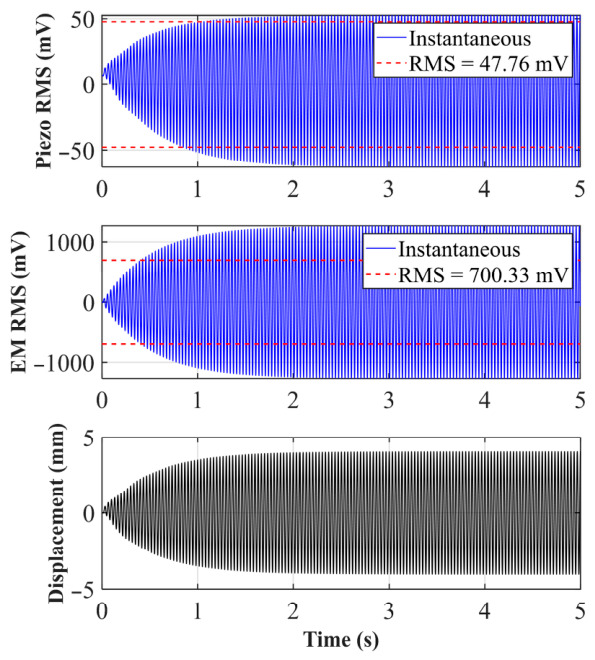
Voltage and displacement of *d*_0_ = 49 mm at 30 Hz.

Due to the slow increase in voltage of the electromagnetic unit in the 0 Hz–20 Hz sweep simulation in Figure 8, and the absence of peak phenomena, time-domain simulation analysis was conducted at 25 Hz and 30 Hz, as shown in Figure 9 and Figure 10. From the simulation results, it can be seen that there is still an upward trend in these three frequencies, and 30 Hz is far beyond the frequency that can be generated by human motion. Therefore, when *d*_0_ = 49 mm, it cannot be used for harvesting human energy.

In summary, the nonlinear changes and fluctuations in voltage waveforms with narrow spacing (such as 25 mm, 30 mm) are very severe, resulting in extremely high voltage output. The voltage waveform with a wide spacing (such as 40 mm) is more rounded (close to a sine wave), and although the voltage value decreases, the system can still operate stably at low frequencies (around 9.3 Hz). Due to the extremely weak magnetic coupling, the voltage waveform fluctuation is not obvious, and the displacement is small, resulting in low electromagnetic and piezoelectric voltages (49 mm), which proves the importance of magnetic coupling from the opposite side.

### 3.2. Simulation Analysis of the Influence of Coil Turns on Electromagnetic Voltage

This section designs four types of turns, namely 200 turns, 400 turns, 600 turns, and 800 turns, with an initial distance of *d*_0_ = 40 mm. Because the number of coil turns almost does not affect the mechanical force on the piezoelectric unit, this section will peel off the piezoelectric unit and focus on exploring the characteristics of electromagnetic energy conversion in the system due to different numbers of turns.

**Figure 11:**
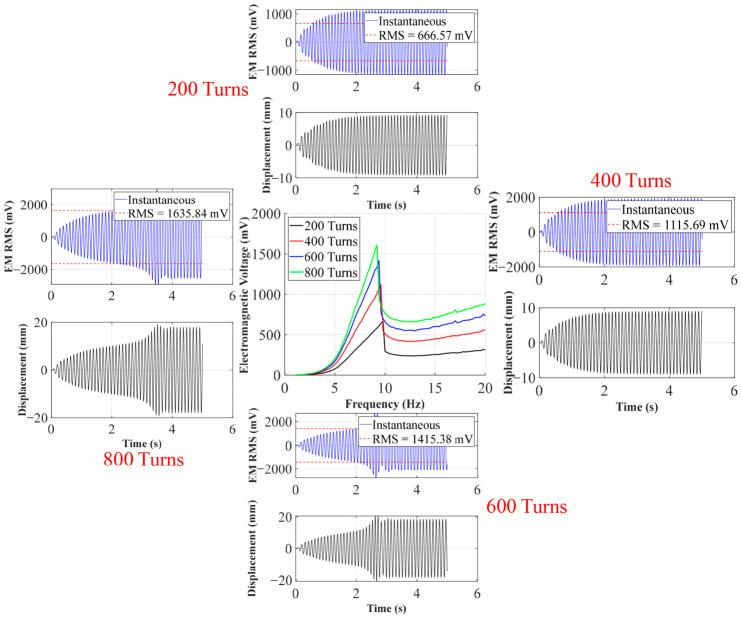
Simulation of electromagnetic voltage with different numbers of turns and peak frequencies.

As shown in Figure 11, as the coil gradually increases from 200 turns to 800 turns, the overall electromagnetic voltage exhibits a significant step-like upward trend. For example, at 200 turns, its peak voltage is only 666.57 mV, but when it increases to 600 turns and 800 turns, the peak voltages reach 1415.4 mV and 1635.8 mV, respectively. According to Faraday’s law of electromagnetic induction, the open-circuit induced electromotive force generated by a system at the same mechanical amplitude is proportional to the number of coil turns. The increase in the number of turns directly amplifies the electromechanical coupling coefficient of the system *Θ*, allowing the magnet to cut more magnetic induction lines when passing through the coil, thus visually representing the overall elevation of electromagnetic voltage on the sweep frequency diagram.

In order to observe the motion state and output voltage under different turns in depth, peak frequencies corresponding to each turn (such as 9.8 Hz at 200 turns, 9.4 Hz at 600 turns, etc.) were extracted for time-domain simulation. From the time-domain waveform, it can be seen that although the increase in the number of turns brings about a significant increase in voltage, the maximum mechanical displacement of the central magnet in steady state shows a slight suppression trend. This is because, as the number of turns increases, the electromagnetic damping force that hinders the displacement of the central magnet also significantly increases.

### 3.3. Simulation Analysis of the Influence of Central Magnet Mass on Piezoelectric Voltage

This section designs four different masses of magnets, namely 2 g, 2.4 g, 3 g, and 4 g, with a coil turn count of 800 and an initial distance of *d*_0_ = 40 mm. In a hybrid energy harvesting system, the central magnet not only serves as a magnetic induction source that generates induced electromotive force but also as a mass that causes small deformations in the piezoelectric sheet. Due to the constant magnetic field strength, in order to better explore the influence of mass on piezoelectric energy conversion, this section will strip off the electromagnetic unit and focus on exploring the influence of the central magnet mass on the piezoelectric energy conversion characteristics of the system.

**Figure 12:**
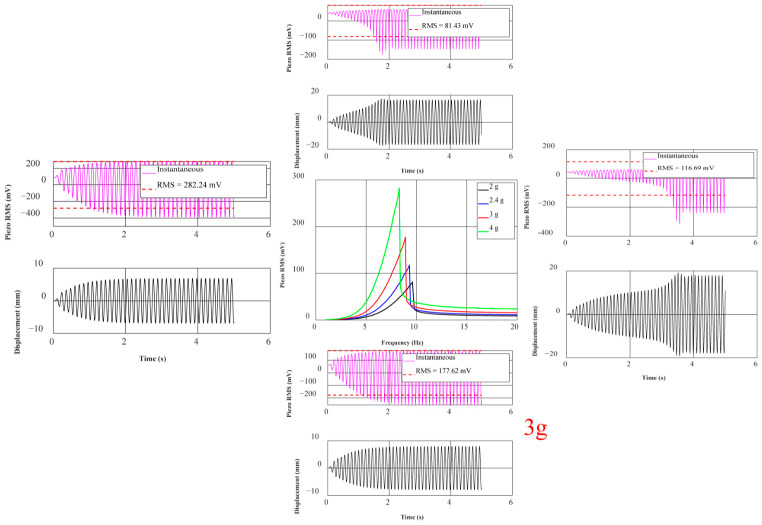
Simulation of piezoelectric voltage with different masses, sweep frequencies and peak frequencies.

As shown in the simulation results in Figure 12, the mass of the magnet has a dual effect on the system’s resonant response. First, a frequency redshift occurs; as the magnet mass increases from 2 g to 4 g, the system’s resonant peak frequency exhibits the classical mechanical vibration behavior ($\omega_{n}\propto m^{-1/2}$), decreasing from 9.6 Hz to 8.3 Hz. Second, as the magnet mass increases, the effect of gravitational acceleration on the piezoelectric unit becomes more significant, and the voltage correspondingly increases gradually from 81.4 mV to 282.2 mV. This nonlinear growth in voltage shows excellent performance.

However, although larger magnets (4 g) enable the system to have greater voltage output capability at low frequencies, with voltages up to nearly 300 mV, the excessive displacement they may cause can easily lead to destructive rigid collisions between the magnet and the arched plate, and also keep the piezoelectric layer under extreme strain cycles for long periods, significantly shortening the fatigue life of the piezoelectric sheet. Overall, 2.4 g magnets limit the mechanical amplitude within the structure’s safe range, making them more suitable for harvesting human energy.

## 4. Experimental Research

This experimental protocol was determined directly based on the parameter sensitivity simulations described in Section 3. Simulation results presented in Figure 4, Figure 5, Figure 6, Figure 7, Figure 8, Figure 9 and Figure 10 indicate that the initial distance *d*_0_ governs the magnetic coupling strength and the effective operating bandwidth; Figure 11 demonstrates that the output voltage exhibits a monotonically increasing trend as the number of coil turns increases; and Figure 12 reveals that, as the mass of the central magnet increases, the piezoelectric unit becomes significantly susceptible to gravitational acceleration, thereby having a pronounced impact on the output voltage. Upon completion of the bench-top screening process, the parameter combinations demonstrating superior performance will subsequently be utilized for human motion experiments.

**Figure 13:**
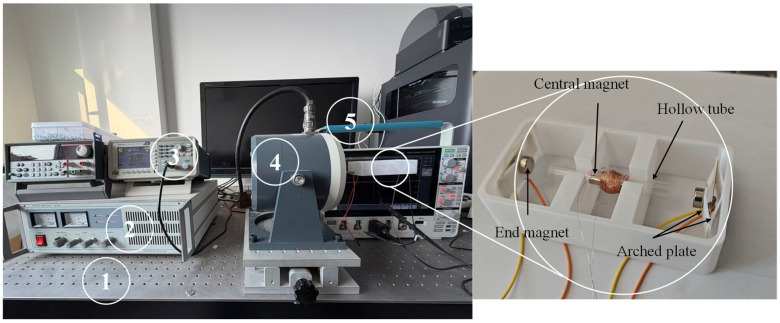
Experimental apparatus.

The experimental setup of the energy harvester is shown in Figure 13. To ensure measurement accuracy, the PEHEH is mounted on a damping isolation platform (WPD20-10-8, Label 1) to eliminate external environmental vibrations. A signal generator (Tektronix-AFG1022, Label 3) is utilized to produce a precise input electrical signal, which is subsequently amplified by a power amplifier (GF 200, Label 2) to drive the electromagnetic exciter (JZQ-20, Label 4). The exciter thus provides the controlled mechanical vibration to the harvester. Finally, the real-time output voltages generated by the piezoelectric and electromagnetic units were measured separately and analyzed using a digital oscilloscope (Tektronix-MDO4024C, Label 5. Tektronix, Shanghai, China). For the electromagnetic measurement, the probe tip and ground clip were connected to the two ends of the coil, whereas for the piezoelectric measurement, they were connected to the two lead wires of the piezoelectric element. The RMS voltages reported in this work were directly read from the oscilloscope’s built-in RMS measurement function under steady-state operation.

According to theoretical formulas, whether the central magnet can achieve reciprocating motion depends on the combined effect of magnetic coupling and external excitation. Therefore, the energy harvesting performance is analyzed from two aspects: material parameters and external excitation. The analysis parameters of the proposed energy harvester are shown in Table 1.

### 4.1. Experimental Investigation of Output Characteristics Under Different Parameters

**Figure 14:**
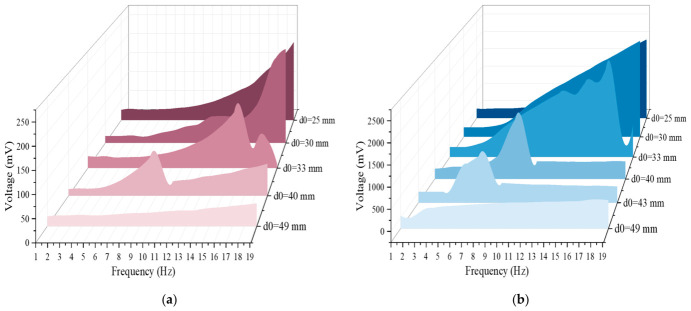
Effect of voltage at initial distance *d*_0_. (**a**) Piezoelectric voltage at different initial distances, increasing with frequency; (**b**) Electromagnetic voltage at different initial distances, increasing with frequency.

Figure 14a shows the RMS output voltage of a single-side piezoelectric layer at different initial distances (the voltages in the following refer to RMS voltages). It can be seen that the magnitude of the initial distance *d*_0_ has a significant impact on the output voltage: When the initial distance *d*_0_ = 30 mm, the output voltage is the maximum, and the single-side voltage can reach 248 mV at the maximum. This is because when the initial distance is small, the displacement of the central magnet is limited, and with the increase in frequency, it can produce a large acceleration, so the force applied to the piezoelectric layers on both sides is also larger. However, the initial distance is not as small as possible. When *d*_0_ = 25 mm, the output voltage drops significantly, and the displacement of the central magnet is limited to a certain range to impose less force on the piezoelectric layers on both sides, which affects the output voltage. With the increase in initial distance, when *d*_0_ is equal to 33 mm, 40 mm and 49 mm, respectively, the voltage decreases significantly. Especially when *d*_0_ = 49 mm, the excitation externally applied is not enough to make the central magnet reciprocate greatly, and the magnetic force applied to the piezoelectric layers on both sides is small, so the magnetic coupling is weak, and the output voltage is small. In addition, when the initial distance is less than or equal to 30 mm, the voltage increases with the increase in frequency due to the small initial distance. When the initial distance is more than 30 mm, the voltage reaches the peak value at a certain frequency and then drops rapidly. To sum up, the initial distance is not linearly related to the piezoelectric layer voltage, but it has a nonlinear trend of increasing first and then decreasing.

Figure 14b shows the voltage output by the electromagnetic unit under different initial distances *d*_0_. It can be seen from the figure that the impact of the initial distance on the voltage of the electromagnetic unit is also significant: when *d*_0_ = 30 mm, its peak value can reach 2500 mV, and similar to the situation of the piezoelectric unit, the initial distance *d*_0_ is not necessarily better when smaller. When *d*_0_ = 25 mm, the output voltage peak value is reduced by about 500 mV compared with the former. As the initial distance *d*_0_ is very small, the displacement of the central magnet is also very small. Restricted by the magnetic repulsion force of the end magnets on both sides, it can only move within a very small range. However, with the gradual increase in frequency, even with the displacement within a very small range, the number of times passing through the coil in unit time is also very large, and the change rate of the magnetic flux is very large. Therefore, the voltage of the initial distance *d*_0_ at or below 30 mm (but not more than 20 mm) is positively related to the frequency. Limited by the experimental conditions, when *d*_0_ is less than or equal to 20 mm, the generated voltage cannot be measured because the initial distance *d*_0_ is too small. When *d*_0_ is very small, although the magnetic flux gradient is larger, the displacement of the system will be limited due to the strong magnetic repulsion force, even if the friction state changes and the movement is limited, finally resulting in the speed amplitude reduction; the voltage is not necessarily larger, and may drop or saturate. It should be noted that the approximate monotonic rising trend at *d*_0_ = 25 mm and *d*_0_ = 30 mm seems to increase with the increase in frequency, but the real reason is that the frequency around 19 Hz does not reach the real upper limit of voltage generated by the energy harvester; in contrast, when the initial distance *d*_0_ = 33 mm and *d*_0_ = 40 mm, there is a phenomenon of jumping, which rises first and then drops with the increase in *d*_0_. In addition, when *d*_0_ increases from 33 mm to 40 mm, the magnetic flux gradient and magnetic coupling are weakened, and the frequency band and peak position of the nonlinear effect will shift, and the peak amplitude tends to decrease, but the hopping feature is still retained. When *d*_0_ = 49 mm, the full-band output is low, and the change is gentle. Too large initial distance *d*_0_ leads to too small change rate of magnetic flux and too weak coupling between magnet and coil, so energy conversion cannot be effectively carried out.

**Figure 15:**
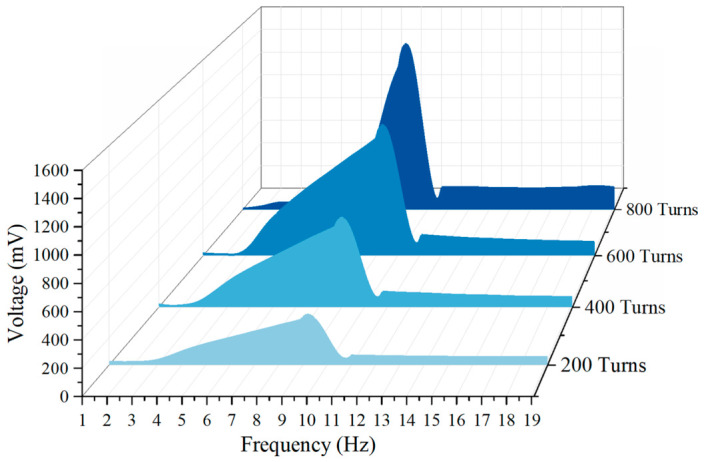
Effect of coil turns on electromagnetic voltage.

In this section, we take *d*_0_ = 40 mm as an example to discuss the influence of the number of turns on the electromagnetic voltage with the increase in frequency. From Figure 15, it can be seen that the voltage fluctuation is not obvious in chaotic state at low frequency band (less than 3 Hz), and the voltage of each curve fluctuates between 14 mV and 25 mV, with no obvious difference, which indicates that the relative motion speed between magnet and coil is small at this time, and the change rate of magnetic flux is not enough to produce obvious induced voltage. In the rising section (3 Hz~9 Hz), the voltage increases obviously, the output voltage increases rapidly with the frequency, and the more the number of turns is, the more obvious the increase is. In the peak section (9 Hz~10 Hz), there is an obvious peak, and the peak value increases significantly with the increase in the number of turns.

The peak value of 200 turns is about 375 mV, the peak value of 400 turns is about 715 mV, the peak value of 600 turns is about 1090 mV, and the peak value of 800 turns is about 1510 mV. The peak value and the number of turns show an approximate linear growth characteristic. The 200 → 400 turns are basically doubled, and the 200 → 800 turns are about four times, which indicates that the impact caused by the increase in the number of turns in this working area is significant. The post-peak attenuation and platform end are in the range of 10 Hz–19 Hz: after the peak near 10 Hz, the voltage drops rapidly and then enters the relatively flat platform area in the higher frequency band. The voltage in the platform area still rises as the number of turns increases, but the increase is not as obvious as that near the resonance.

Increasing the number of turns increases the voltage because the electromagnetic induced voltage meets the basic relationship

(6) $$V=N\frac{d\Phi }{dt}$$

where *N* is the number of turns. Increasing the number of turns *N* will directly increase the induced voltage without changing other conditions. Therefore, the overall increase of 200 turns → 800 turns voltage in the figure is a compound electromagnetic induction law.

**Figure 16:**
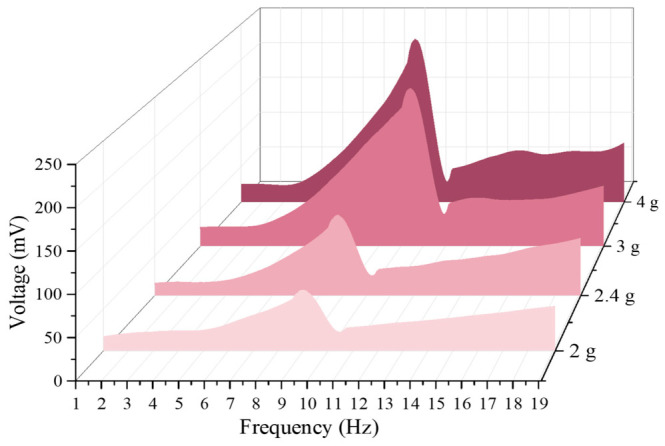
Trend chart of voltage (single side) with different mass, increasing with frequency.

As shown in Figure 16, under the condition of the initial distance *d*_0_ and a constant friction coefficient, the output voltage varies with frequency for different masses. In the low-frequency range of 1 Hz to 4 Hz, the voltage outputs are close to each other with little difference, and the distinction between the outputs of each magnet is not strong, indicating that the system has not yet entered a significant, strong, nonlinear working region. In the mid-frequency range of 4 Hz to 11 Hz, the voltage rises rapidly, and the larger the mass, the steeper the rise. Around 4.5 Hz, all four curves enter a significant growth region, and the peak value increases significantly with the mass of the magnets, from about 81 mV (9.6 Hz) for a 2 g magnet to about 240 mV (9.9 Hz) for a 4 g magnet. With increased magnet mass, the system is more likely to achieve larger displacement and stronger force transmission, causing greater stress on the piezoelectric layer and increasing the output. Near 10 Hz, after reaching the peak, the voltage drops sharply. This ‘post-peak sudden drop’ pattern is a jumping characteristic of nonlinear systems. In the high-frequency range of 11 Hz to 19 Hz, the voltage is generally lower, then it gradually recovers and tends to converge, but the overall output voltage of the larger 3 g and 4 g magnets is still higher than that of the 2 g and 2.4 g magnets. By comparison, it is found that the mass of the central magnet significantly affects the voltage output of the piezoelectric unit. The peak voltage increases significantly with the magnet mass, rising from 81 mV to 240 mV at most.

Through the comparison between the third section and the fourth section, it is found that the trend and output voltage characteristics obtained by simulation and experiment are relatively consistent, so the correctness of the derived formula is validated, and the correctness of the theoretical simulation results is also verified, which provides strong support for the following human energy harvesting.

### 4.2. Comparison Between Experimental and Simulation Results

**Figure 17:**
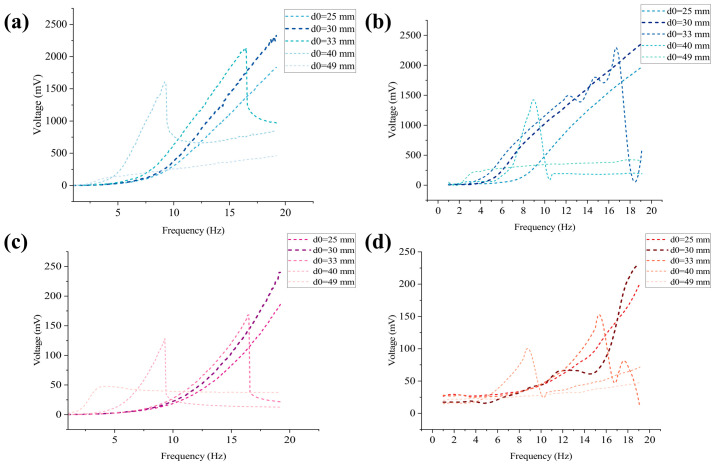
Comparison between simulation and experiment for the effect of initial distance: (**a**) Simulation: electromagnetic voltage; (**b**) Experiment: electromagnetic voltage; (**c**) Simulation: piezoelectric voltage; (**d**) Experiment: piezoelectric voltage.

Figure 17 compares the simulation and experimental results for the effect of initial distance on the piezoelectric and electromagnetic voltages. The two sets of results show the same overall trend: the initial distance strongly affects both outputs, and the voltage does not change linearly with *d*_0_. In both cases, an intermediate initial distance gives better performance than an excessively small or large distance. A difference can still be observed in the optimal value; the best result appears at *d*_0_ = 40 mm in the simulation but at *d*_0_ = 30 mm in the experiment. This difference is likely caused by factors not fully included in the model, such as fabrication error, assembly deviation and friction variation.

**Figure 18:**
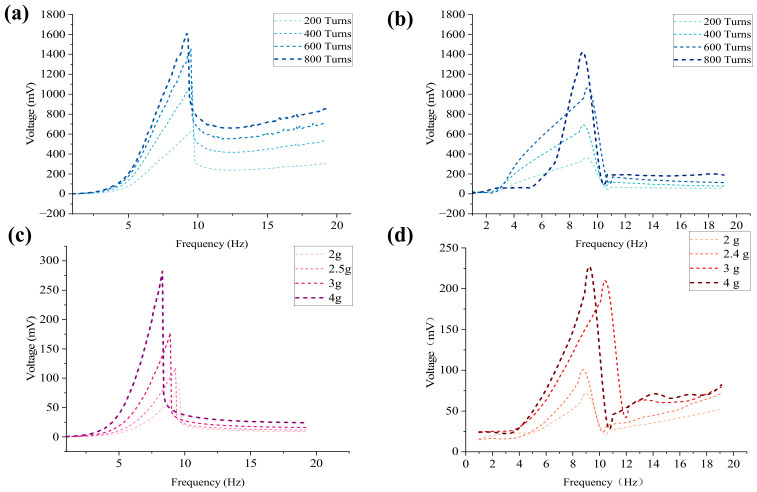
Comparison between simulation and experiment for the effects of coil turns and central magnet mass. (**a**) Simulation: effect of coil turns on electromagnetic voltage; (**b**) Experiment: effect of coil turns on electromagnetic voltage; (**c**) Simulation: effect of central magnet mass on piezoelectric voltage; (**d**) Experiment: effect of central magnet mass on piezoelectric voltage.

Figure 18 compares the simulation and experimental results for the effects of coil turns and central magnet mass. For the electromagnetic unit, both the simulation and the experiment show that the output voltage increases as the number of coil turns increases. The same overall trend is observed in both cases, indicating that the model harvests the main effect of coil turns on electromagnetic output. For the piezoelectric unit, both simulation and experiment show that increasing the mass of the central magnet leads to a higher output voltage within the tested range. This agreement further supports the validity of the model and its ability to describe the main response of the PEHEH under different parameter settings. Although small differences can still be observed in the peak position and local curve shape, the overall agreement between simulation and experiment remains good.

Overall, the experiments agree well with the simulations in their main trends. The results confirm that the model captures the key behavior of the PEHEH despite small differences in the optimal values and local curve details.

## 5. Human Motion Experiment

**Figure 19:**
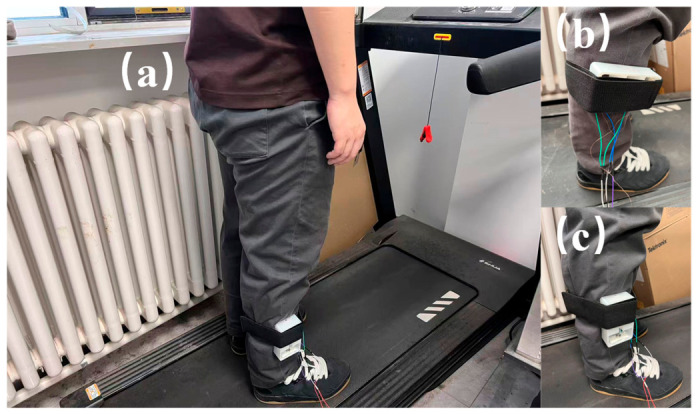
Experiment mode of human motion energy harvesting. (**a**) Overall view; (**b**) Horizontal fixed view; (**c**) Vertical fixed view.

In order to verify the practicality of the PEHEH, the harvester is fixed on the human leg, and the output voltage of the harvester under different conditions is studied and compared. The experiment was conducted on a treadmill to test the voltage output characteristics of electromagnetic and piezoelectric units under the three states of slow walking, fast walking and slow running. As mentioned above, when *d*_0_ = 30 mm, the number of turns is 800 to test human body motion. As shown in Figure 19, during the experiment, the harvester was fixed on the lower leg of an experimenter (male), who is 170 cm in height and 75 kg in weight, and it measured the output voltage of the harvester under different motion speeds. The output voltage of the harvester is recorded by an oscilloscope, and each test is 20 s.

When the human body is walking, the acceleration generated by the leg is decomposed into acceleration in the toe direction and acceleration in the tibial direction. Therefore, in this paper, the horizontal and vertical installation methods are respectively adopted for energy harvesting during the swing of the leg.

When fixed horizontally, the central magnet moves in the direction of the toe, as shown in Figure 19b; when the magnet is fixed vertically, the movement direction of the central magnet is the tibial axis direction, as shown in Figure 19c. Due to different physical functions of men and women, under the same movement speed, the pace and acceleration excitation intensity vary from person to person, so the test results may be different for different experimenters with different physical parameters.

**Figure 20:**
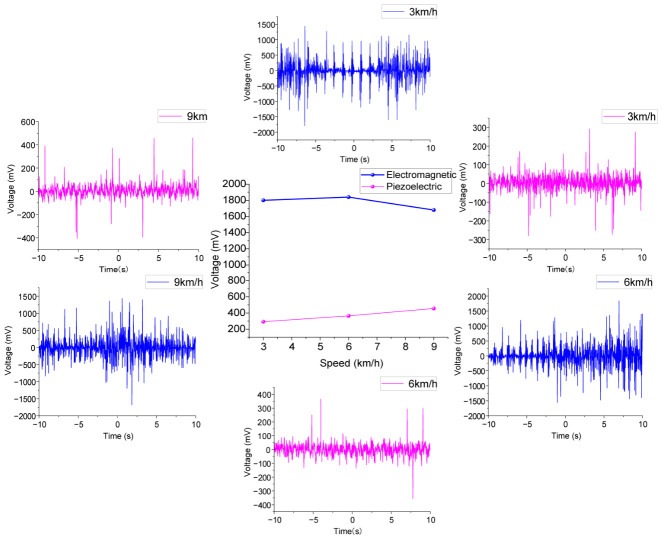
Voltage response of piezoelectric and electromagnetic units at horizontal fixation.

To study the influence of motion speed on the output of PEHEH, the experimenter conducted experiments at different motion speeds on the treadmill, where 3 km/h, 6 km/h and 9 km/h correspond to slow walking, fast walking and slow running, respectively. Figure 20 shows the voltage response of the harvester horizontally fixed to the experimenter’s lower leg at speeds of 3 km/h, 6 km/h and 9 km/h. For the slow state, the maximum voltage generated by the piezoelectric unit is 292 mV, and the maximum voltage generated by the electromagnetic unit is 1.8 V; for the fast state, the maximum voltage generated by the piezoelectric unit is 364 mV, and the maximum voltage generated by the electromagnetic unit is 1.84 V; and for the slow running state, the maximum voltage generated by the piezoelectric unit is raised to 456 mV, and the maximum voltage generated by the electromagnetic unit is 1.68 V. For the piezoelectric unit, different walking states have a significant impact on the piezoelectric voltage. As the speed becomes faster, the piezoelectric unit is sensitive to the impact of gravity acceleration. From the slow walking state to the fast walking state, the piezoelectric voltage increases by about 25%; when the electromagnetic unit is fixed horizontally, it is not sensitive to the change in external excitation. Even when the motion speed reaches 9 km/h, the voltage shows a downward trend. Although the higher step frequency increases the time frequency of the magnetic flux change, the jogging posture changes the swing trajectory and impact direction of the lower leg, resulting in limited displacement of the central magnet in the hollow tube and thus reducing the voltage.

**Figure 21:**
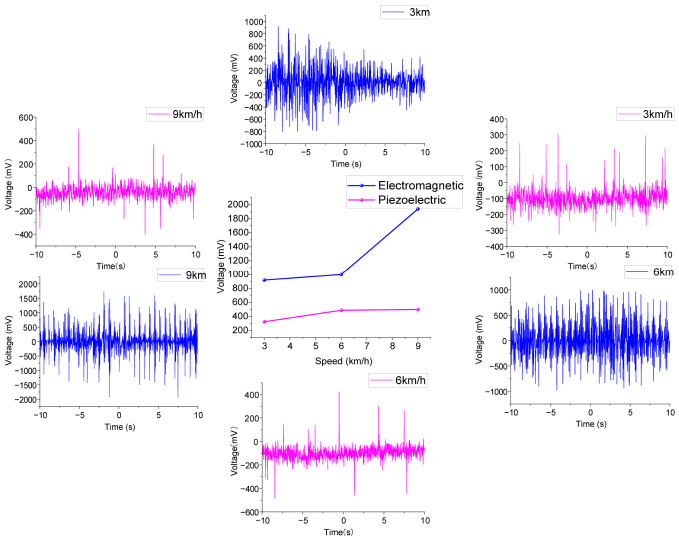
Voltage response of piezoelectric and electromagnetic units in vertical fixation.

Figure 21 shows the voltage response of the harvester vertically fixed to the experimenter’s lower leg at speeds of 3 km/h, 6 km/h and 9 km/h. When traveling at a speed of 3 km/h, the maximum voltage output by the piezoelectric unit is 324 mV, and the maximum voltage output by the electromagnetic unit is 0.92 V; when traveling at a speed of 6 km/h, the maximum voltage output by the piezoelectric unit is 488 mV, and the maximum voltage output by the electromagnetic unit is 1 V; and when the speed increases to 9 km/h, the maximum voltage output by the piezoelectric unit is 500 mV, and the maximum voltage output by the electromagnetic unit is 1.94 V. The voltage of the piezoelectric unit increases from 324 mV to 500 mV, which shows that the intense movement along the tibial axis of the human body, especially the impact energy of feet touching the bottom during jogging, can be effectively converted into the vertical inertia force of the central magnet, forcing the piezoelectric layer to output higher voltage; the electromagnetic unit increases from 0.92 V to 1.94 V. Due to the superposition effect of the vertical acceleration and gravity of the human body, the reciprocating displacement of the central magnet is greatly increased, making the cutting speed of the magnetic induction line faster. It should be noted that, due to different fixing modes, the electromagnetic unit and the piezoelectric unit on both sides are more affected by the acceleration of gravity during vertical fixing, so the output voltage is slightly greater than that of horizontal fixing.

## 6. Conclusions

This paper proposes a dual magnetically coupled low-frequency PEHEH. The PEHEH generates an induced electromotive force through a coil with a central magnet and utilizes magnetic repulsion to apply force to the piezoelectric layer via an arched plate, thereby outputting voltage. Dynamic simulations reveal that increasing the mass of the magnet and altering the initial magnetic spacing can effectively trigger the redshift of the system’s resonant frequency and nonlinear amplification of dynamic displacement, significantly enhancing the overall energy harvesting efficiency in low-frequency environments. The experimental study shows that when the coil turns are 800 and the value of *d*_0_ = 30 mm, the electromagnetic unit can output a large voltage, the maximum output voltage of the electromagnetic unit can be 2.5 V, and the maximum output voltage of the piezoelectric unit can be 248 mV. In human experiments, the maximum output voltage of the electromagnetic unit can reach 1.94 V, and the output voltage of the piezoelectric unit can reach 500 mV.

The output voltage amplitude of the energy harvester is significantly related to the mass of the central magnet, the initial distance and the number of coil turns and has different output characteristics under different conditions. In future research, we will further optimize the harvester structure, quantify the energy loss caused by eccentric contact and local tilting, and investigate the measurement and power-management circuits to further improve energy harvesting efficiency.

## Figures and Tables

**Table 1:** Parameter values for the piezoelectric and electromagnetic modules.

Parameter	Value	Parameter	Value
Material of arched plate	PLA	Piezoelectric layer material	PZT-5H
The length of the bonding plane $l_{1}$	5	Piezoelectric constant $d_{31}$ ($10^{-10}\;{\mathrm{CN}}^{-1}$)	−3.2
The length of the inclined plane $l_{2}$	12.9	Piezoelectric constant $d_{33}$ ($10^{-10}\;{\mathrm{CN}}^{-1}$)	6.5
Length of the upper plane $l_{3}$	7	Piezoelectric layer thickness $t_{p}$ (mm)	0.5
Included angle θ (°)	15	Elastic compliance of piezoelectric layer $s_{11}$ ($10^{-11}\;m^{2}N^{-1}$)	1.65
Width of arched plate b	10	Equivalent capacitance of piezoelectric layer $C_{p}$ ($10^{-9}\;F$)	6.25
Arched plate $t_{m}$ thickness (mm)	0.5	Magnet material	NdFeB
Modulus of elasticity of arched plates $E_{m}$ (Gpa)	3.1	Mass of central magnet $M_{c}$ (kg)	0.005
Poisson’s ratio of arched plate $v_{m}$	0.35	Magnetic moment M (Am^2^)	0.1
Electromagnetic damping coefficient $D_{c}\;(N\cdot s/m)$	0.005	Coefficient of sliding friction λ	0.05
Electromagnetic coupling coefficient *Θ* (Wb/m)	1.2	The width of the arched plate b (mm)	15

## Data Availability

Data is contained within the article.

## Abbreviations

The following abbreviations are used in this manuscript:

PEHEH	Piezoelectric–electromagnetic Hybrid Energy Harvester
